# Edentulism, severe tooth loss, and lack of functional dentition in elderly people in Brazil: prevalence and social inequalities in the 2023 National Oral Health Survey

**DOI:** 10.1590/1980-549720260023.supl.1

**Published:** 2026-07-24

**Authors:** Renato Canevari Dutra da Silva, Heloísa Silva Guerra, Fernanda de Oliveira Meller, Cleidiane Aparecida de Quadra, Elton Brás Camargo, Antônio Augusto Schäfer

**Affiliations:** I Universidade de Rio Verde, School of Dentistry, Rio Verde Campus, Research Group on Epidemiology and Health - Rio Verde (GO), Brazil.; II Universidade de Rio Verde, School of Medicine, Goiânia Campus, Research Group on Epidemiology and Health - Goiânia (GO), Brazil.; III Universidade do Extremo Sul Catarinense, Graduate Program in Collective Health - Criciúma (SC), Brazil.; IV Universidade de Rio Verde, Vice-rector of Post-Graduation, Research Group on Epidemiology and Health - Rio Verde (GO), Brazil.

**Keywords:** Tooth loss, Aged, Oral health, Social inequity, Epidemiology

## Abstract

**Objective:**

To estimate the prevalence of edentulism, severe tooth loss, and lack of functional dentition among older adults in Brazil and to analyze associated social, racial, and regional inequalities, based on the 2023 National Oral Health Survey.

**Methods:**

A population-based cross-sectional study was conducted with individuals aged ≥65 years. Outcomes included edentulism, severe tooth loss (0-8 teeth), and lack of functional dentition (<21 teeth). Associations were estimated using Poisson regression with robust variance.

**Results:**

A total of 9,746 older adults were included. The prevalence of edentulism was 36.5%, severe tooth loss 48.1%, and lack of functional dentition 72.9%. In the adjusted analysis, women showed higher prevalence of all outcomes, which also increased with age. Low educational level was the main factor associated with worse oral health conditions: individuals with no schooling had higher prevalence of edentulism (PR=3.62; 95%CI: 2.48-5.30) and severe tooth loss (PR=3.79; 95%CI: 3.00-5.17), as well as higher prevalence of lack of functional dentition (PR=1.82; 95%CI: 1.52-2.19), compared to those with higher education. Brown-skinned individuals had higher prevalence of severe tooth loss and lack of functional dentition, while living in capital cities was associated with lower prevalence of edentulism.

**Conclusion:**

Edentulism, severe tooth loss, and lack of functional dentition remain strongly associated with social, racial, and regional inequalities in Brazil. These findings highlight the need for public policies focused on oral health equity, emphasizing prevention, prosthetic rehabilitation, and expanded access to services for vulnerable populations.

## INTRODUCTION

The condition of dentition constitutes an important indicator of oral health, reflecting the cumulative history of exposure to risk factors, illness, and access to dental services throughout life. Changes in dentition influence aesthetic, nutritional, and psychosocial aspects, impacting chewing, speech, self-esteem, and individuals’ quality of life^1^ ^,^ ^2^. Despite the global reduction in the prevalence of dental alterations in recent decades, challenges remain in low- and middle-income countries, where social determinants continue to shape the unequal distribution of these outcomes^3^ ^,^ ^4^.

Edentulism, characterized by the complete lack of teeth, is an important public health problem, as it reflects the accumulated burden of oral diseases and profound social inequalities^5^. In different Brazilian contexts, studies indicate prevalences ranging from 46.4% in municipal household samples to more than 62% in institutionalized populations, suggesting a possible stagnation in the decline observed in previous decades among specific subgroups^6^ ^,^ ^7^ ^,^ ^8^.

In addition to edentulism, intermediate conditions involving a reduced number of teeth function as sensitive markers of oral health trajectories. The literature highlights that maintaining a functional dentition, often defined as 20 or 21 teeth, is essential to reduce negative impacts on quality of life, although functional indicators still vary across national studies^9^ ^,^ ^10^. The number of remaining teeth is unevenly distributed, reflecting regional and socioeconomic differences and inequalities in access to preventive and restorative care^11^ ^,^ ^12^ ^,^ ^13^ ^,^ ^14^.

Individuals with lower levels of education and income, belonging to racial minority groups, residing in rural areas or in less developed regions, and women are more likely to present significant dental alterations^1^ ^,^ ^6^ ^,^ ^12^. However, a persistent gap is noted in the literature prior to the survey conducted by the National Oral Health Survey (SB Brasil) 2023 regarding the in-depth analysis of racial disparities and the integration of service provision data to explain the observed regional variations, since most of the available evidence comes from local or convenience samples with limited generalizability^15^ ^,^ ^16^.

The National Oral Health Policy - Brasil Sorridente - expanded access to preventive and restorative care, in addition to offering prosthetic rehabilitation; however, challenges related to coverage, continuity of care, and overcoming regional and social inequalities persist^17^ ^,^ ^18^ ^,^ ^19^.

The national epidemiological survey SB Brasil constitutes the main source of data on the oral health conditions of the Brazilian population. Its previous editions (2003 and 2010) highlighted marked regional and socioeconomic inequalities in indicators such as tooth loss and edentulism^20^ ^,^ ^21^.

Given the more than decade-long gap in the most comprehensive national data, SB Brasil 2023 is essential to update the oral health landscape in the country and overcome limitations of fragmented studies. Thus, this study aimed to estimate the prevalence of edentulism, severe tooth loss, and lack of functional dentition in individuals aged 65 years or older, as well as to assess the social, racial, and regional inequalities associated with these outcomes, based on representative data from SB Brasil 2023.

## METHODS

### Study design

This is a cross-sectional population-based study using data from SB Brasil 2023, coordinated by the Ministry of Health. SB Brasil is the main national epidemiological oral health survey, with probabilistic multistage sampling. In this study, the elderly population (≥ 65 years) was analyzed in order to assess outcomes related to tooth loss and the maintenance of functional dentition.

### Population and sampling

The SB Brasil 2023 sampling plan was designed to ensure national and regional representativeness, as well as representation for state capitals and inland areas. Selection occurred in three stages: census tracts, drawn based on the National Address Register for Statistical Purposes of the Brazilian Institute of Geography and Statistics (CNEFE/IBGE); households, systematically selected within each tract; and eligible individuals, invited according to the survey’s index ages (5, 12, 15-19, 35-44, and 65-74 years). In this study, all participants aged 65 years or older were included, totaling 9,746 older adults.

### Data collection

The data collection took place between 2022 and 2023 by teams composed of dentists and annotators, previously trained and calibrated. The process included theoretical activities, virtual exercises, and in-person stages, with analysis of photographs and standardized clinical examinations.

Agreement with the gold standard was assessed using the Kappa coefficient, requiring a minimum value of 0.65 for fieldwork participation. The home clinical examinations were conducted under natural lighting, using flat mouth mirrors and sterilized community probes, according to the criteria of the World Health Organization (WHO). A structured questionnaire was also administered with demographic, socioeconomic, access to health services, and oral health condition information, including tooth loss, dental condition, trauma, and treatment needs.

### Studied variables

Three oral health-related outcomes were considered: “edentulism,” defined as the total loss of natural teeth (yes; no); “severe tooth loss,” which is the presence of 0 to 8 remaining teeth (yes; no); and “lack of functional dentition,” defined as the presence of fewer than 21 natural teeth (yes; no). It is important to highlight that the three outcomes studied are not mutually exclusive, and the same individual may fall into more than one category.

The exposure variables included to assess social, racial, and regional inequalities were: sex (male; female), age (in completed years), years of schooling (0; 1-4; 5-8; 9-11; ≥ 12 years), monthly household income in minimum wages (≤ 1; 1-2; 2-3; 3-5; > 5), self-reported skin color (white; black; brown; yellow; Indigenous), macro-region of residence (North; Northeast; Southeast; South; Central-West), municipality (capital city; countryside); receipt of Bolsa Família and/or other government income transfer programs (yes; no).

### Statistical analysis

A descriptive analysis of all studied variables was presented using absolute and relative frequencies, with their respective 95% Confidence Intervals (95%CI).

To assess social, racial, and regional inequalities in the oral health outcomes studied, Pearson’s chi-square test was used, with a 5% significance level, for the crude analyses.

To verify the independence of the associations in relation to potential confounding factors, adjusted analyses were performed using Poisson regression with robust variance, estimating Prevalence Ratios (PR) and their respective 95% confidence intervals (95%CI). The p-values presented correspond to the Wald test for linear trend. The adjusted analysis was conducted using a hierarchical determination model, based on the theoretical framework of the social determinants of health proposed by Dahlgren and Whitehead (1991). Contextual variables related to place of residence (macro-region and municipality) were considered at the distal level. At the intermediate level, individual socioeconomic variables (years of education and monthly income) were included; at the proximal level, demographic characteristics (sex, age, and skin color). The inclusion of variables in the model followed this hierarchical structure, respecting the theoretical gradient of determination between the levels^22^.

All analyses incorporated the sample weight, the effect of the complex survey design, and the sample stratification structure through the `svy` command in Stata 17.0 software (StataCorp, College Station, United States), ensuring correction for the effects of the sampling design.

### Ethical aspects

In compliance with Resolution No. 466, of December 12, 2012, of the National Health Council (CNS), the Project was approved by the National Research Ethics Commission (Conep) on July 3, 2021, under CAAE 34497120.6.3001.0008 and opinion No. 4,823,054. The Informed Consent Form (ICF) was obtained from all participants and legal representatives of vulnerable participants.

## Data Availability Statement:

The entire dataset supporting the results of this study is available upon request from the Ministry of Health: https://www.gov.br/saude/pt-br/composicao/saps/brasil-sorridente/sb-brasil/dados#:~:text=A%20edi%C3%A7%C3%A3o%20mais%20recente%20da%20Pesquisa%20Nacional,a%20porcentagem%20da%20popula%C3%A7%C3%A3o%20brasileira%20livre%20de

## RESULTS

**Figure 1.**
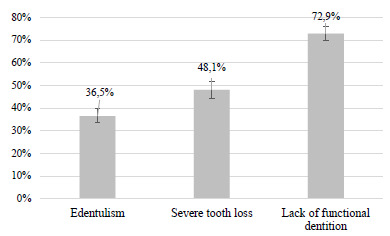
Prevalence of the oral health outcomes studied. SB Brasil, 2023.

The study sample comprised 9,746 individuals aged 65 years or older, the majority of whom were female (61.9%) and nearly half had brown skin color (45.6%). In addition, 37.8% of the older adults had up to 4 years of schooling, and about half of them had a monthly income of up to 2 minimum wages (46.3%). Table 1 and Figure 1 present the description of the sample and the distribution of the prevalence of the outcomes according to demographic and socioeconomic characteristics.

**Table 1.** Description of the sample and distribution of the prevalence of outcomes according to demographic and socioeconomic characteristics. SB Brasil, 2023.

Variables	Total		Edentulism		Severe tooth loss		Lack of functional dentition	
	N	%	%	95%CI	%	95%CI	%	95%CI
Sex (N=9.745)				*p=0.041*		*p=0.038*		*p<0.001*
Male	3,710	38.1	33.4	30.0;37.1	44.9	40.1;49.5	67.6	63.4;71.6
Female	6,035	61.9	38.5	34.4;42.7	50.2	45.7;54.6	76.3	72.7;79.5
Age (N=9,736)				*p<0.001*		*p<0.001*		*p=0.004*
65 years	1,638	16.8	26.7	21.0;33.3	38.6	32.5;45.1	63.4	32.5;45.1
66 years	1,001	10.3	32.8	26.4;40.0	42.4	35.3;49.8	68.8	35.3;49.8
67 years	902	9.3	2.7	20.8;34.0	41.9	34.1;50.1	70.9	34.1;50.1
68 years	932	9.6	36.6	29.6;44.1	47.7	40.1;55.5	71.1	40.1;55.5
69 years	824	8.5	36.9	29.0;45.5	52.7	44.4;60.9	73.1	44.4;61.0
70 years	888	9.1	32.8	26.6;39.7	43.7	36.5;51.3	74.5	36.5;51.3
71 years	672	6.8	44.4	34.4;54.8	57.7	46.7;68.0	84.1	46.7;68.0
72 years	872	9.0	43.0	35.9;50.3	52.1	44.9;59.2	78.6	44.9;59.2
73 years	800	8.2	43.3	34.5;52.5	53.7	45.2;62.0	74.5	45.2;62.1
74 years	1,207	12.4	49.8	42.8;56.9	58.5	51.4;65.2	78.8	51.4;65.2
Skin color (N=9,599)				*p=0.255*		*p=0.006*		*p<0.001*
White	3,601	37.5	34.5	30.0;39.3	44.3	38.9;49.8	67.9	62.7;72.7
Black	1,353	14.1	38.2	32.5;44.3	49.8	43.6;56.0	78.0	72.1;83.0
Yellow	129	1.3	25.5	13.6;42.6	37.8	22.5;56.0	62.1	37.7;81.6
Brown	4,466	46.5	38.7	35.5;42.0	52.2	48.3;56.1	77.4	74.4;80.2
Indigenous	50	0.6	27.0	7.9;61.5	78.4	52.7;92.2	95.7	83.1;99.0
Years of study (N=8,859)				*p<0.001*		*p<0.001*		*p<0.001*
0 year	1,240	14.1	54.1	48.8;59.3	66.3	60.8;71.3	85.5	81.3;88.9
1 to 4 years	2,099	23.7	45.0	39.6;50.6	59.4	52.8;65.7	81.4	75.8;85.9
5 to 8 years	2,484	28.0	38.0	32.1;44.3	50.2	44.5;56.0	78.1	73.9;81.8
9 to 11 years	1,376	15.5	24.2	18.3;31.3	34.7	27.1;43.1	60.5	49.5;70.5
12 years or more	1,660	18.7	13.3	10.2;17.1	17.0	13.6;21.2	43.8	38.1;49.6
Monthly income (minimum wages) (N=9,746)				*p<0.001*		*p<0.001*		*p<0.001*
Up to 1	1,995	20.5	41.2	36.7;45.7	53.0	48.0;57.9	79.6	73.8;84.3
1 to 2	2,510	25.8	37.5	32.6;42.6	50.0	43.9;56.2	75.5	70.2;80.1
2 to 3	1,102	11.3	32.7	26.8;39.2	50.1	41.7;58.5	76.7	71.1;81.4
3 to 5	1,005	10.3	27.3	18.8;37.8	37.2	28.3;47.0	59.8	49.8;69.1
Greater than 5	584	5.9	15.3	10.4;22.0	19.3	13.6;26.8	42.5	32.2;53..5
Not informed	2,550	26.2	41.7	35.6;48.0	51.5	45.5;57.5	75.0	69.4;79.8
Macroregion (N=9,746)				*p=0.579*		*p=0.105*		*p<0.001*
North	2,243	23.0	40.0	35.3;44.8	55.4	49.6;61.2	83.0	77.9;87.2
North East	3,272	33.6	37.6	34.0;41.4	51.9	47.5;56.3	79.8	75.6;83.3
Southeast	1,458	15.0	35.2	29.6;41.4	44.4	37.2;51.8	66.0	60.1;71.4
South	1,425	14.6	35.5	30.2;41.3	48.9	43.6;54.2	77.1	72.0;81.5
Central-West	1,348	13.8	40.5	35.4;45.7	52.3	46.5;58.0	77.4	72.9;81.3
Municipality (N=9,746)				*p=0.032*		*p=0.251*		*p=0.268*
Interior	2,815	28.9	37.8	33.9;41.8	48.9	44.0;53.8	73.5	69.4;77.3
Capital	6,931	71.1	32.4	29.4;35.4	45.5	42.6;48.5	70.8	68.2;73.3
Continuous cash benefit (BPC) (N=9,125)				*p=0.241*		*p=0.149*		*p=0.105*
No	7,772	85.2	35.1	31.7;38.7	46.6	42.4;50.9	71.7	68.2;75.0
Yes	1,353	14.8	39.0	33.5;44.7	51.8	45.8;57.7	77.2	70.8;82.6
Bolsa Família receipt (N=9,190)				*p=0.004*		*p=0.052*		*p<0.001*
No	8,034	87.4	34.6	31.3;38.0	46.5	42.2;50.8	71.3	67.7;74.6
Yes	1,156	12.6	46.3	38.8;54.1	55.7	48.0;63.1	84.3	78.8;88.7
Benefit from another government program (N=9,136)				*p<0.001*		*p<0.001*		*p<0.001*
No	7,999	87.6	33.4	30.3;36.8	45.3	41.3;49.4	71.1	67.5;74.3
Yes	1,137	12.4	49.9	41.3;58.5	60.9	53.4;68.1	81.8	76.0;86.5

CI: confidence interval; Pearson’s chi-square test.

The prevalence of edentulism was 36.5%, being more prevalent among women (38.5%) compared to men (33.4%; p=0.041). Similarly, severe tooth loss was observed more frequently among women (50.2% versus 44.9%; p=0.038), as was the lack of functional dentition, which also showed significant differences according to sex (76.3% female versus 67.6% male; p<0.001).

The prevalence of edentulism and severe tooth loss increased progressively with age. Individuals aged 65 years had 26.7% of edentulism and 38.6% severe tooth loss, while those aged 74 years reached 49.8% and 58.5%, respectively (p<0.001). The lack of functional dentition followed a Similar pattern, ranging from 63.4% at 65 years to 78.8% at 74 years (p=0.004).

Skin color was also associated with two evaluated outcomes. Severe tooth loss was more frequent among black individuals (49.8%) and mixed-race individuals (52.2%) compared to white individuals (44.3%; p=0.006), and the lack of functional dentition followed a similar trend, being higher among black (78.0%) and mixed-race (77.4%) individuals compared to white individuals (67.9%; p<0.001).

Years of education showed an inverse association with all outcomes. Individuals with no schooling had prevalences of edentulism, severe tooth loss, and lack of functional dentition of 54.1%, 66.3%, and 85.5%, respectively, while those with 12 or more years of education presented significantly lower values (13.3%, 17%, and 43.8%; p<0.001). Similarly, monthly income was also inversely associated with the outcomes: individuals earning up to one minimum wage showed a higher prevalence of edentulism (41.2%), severe tooth loss (53%), and lack of functional dentition (79.6%), whereas those with an income above five minimum wages presented significantly lower prevalences (15.3%, 19.3%, and 42.5%; p<0.001).

The lack of functional dentition was more frequent in the North (83%) and Northeast (79.8%) regions, compared to the other macro-regions of the country (p<0.001). At the municipal level, edentulism was more prevalent among residents of inland areas (37.8%) than among those living in the capital city (32.4%; p=0.032).

Individuals benefiting from government programs showed higher prevalences across all three outcomes compared to those who did not receive benefits. Among Bolsa Família beneficiaries, 46.3% presented edentulism versus 34.6% among non-beneficiaries (p=0.004), while the lack of functional dentition was 84.3% versus 71.3% (p<0.001). Similar results were observed among beneficiaries of other government programs (p<0.001 for all outcomes) (Table 1 and Figure 1).

After adjustment for possible confounding factors, most associations were maintained, and the results are presented in Table 2. A higher prevalence of edentulism was found among women (PR=1.19; 95%CI 1.04-1.36) and older elderly individuals, reaching the highest prevalence at 74 years of age (PR=1.96; 95%CI 1.51-2.52). Elderly individuals with no schooling (0 years of education) were 3.62 times more likely to have edentulism than those with higher education (12 years or more) (PR=3.62; 95%CI 2.48-5.30). In addition, individuals living in the country’s state capitals were less likely to have edentulism (PR=0.86; 95%CI 0.74-0.99).

**Table 2.** Adjusted analysis* of the association between outcomes and demographic and socioeconomic characteristics. SB Brasil, 2023.

Variables	Edentulism		Severe tooth loss		Lack of functional dentition	
	RP (95%CI)	p-value	RP (95%CI)	p-value	RP (95%CI)	p-value
Sex		0,002		0.001		< 0.001
Male	Reference		Reference		Reference	
Female	1.22 (1.08;1.40)		1.24 (1.09;1.41)		1.20 (1.11;1.29)	
Age (years)		< 0,001**		0.012		< 0.001**
65	Reference		Reference		Reference	
66	1.26 (0.93;1.72)		1.13 (0.91;1.40)		1.15 (0.94;1.41)	
67	1.04 (0.74;1.47)		1.13 (0.89;1.44)		1.24 (1.06;1.46)	
68	1.30 (1.01;1.67)		1.20 (1.00;1.46)		1.10 (0.96;1.25)	
69	1.37 (0.96;1.95)		1.32 (1.08;1.61)		1.15 (0.98;1.35)	
70	1.11 (0.80;1.55)		0.99 (0.76;1.29)		1.16 (0.98;1.38)	
71	1.56 (1.17;2.08)		1.31 (1.02;1.68)		1.37 (1.21;1.56)	
72	1.54 (1.15;2.05)		1.30 (1.07;1.57)		1.25 (1.10;1.42)	
73	1.49 (1.06;2.10)		1.45 (1.12;1.85)		1.29 (1.10;1.50)	
74	1.62 (1.23;2.14)		1.33 (1.08;1.64)		1.24 (1.08;1.43)	
Skin color		0,681		0.037		0.037
White	Reference		Reference		Reference	
Black	0.97 (0.81;1.18)		0.94 (0.78;1.12)		1.06 (0.93;1.22)	
Yellow	0.64 (0.36;1.14)		0.80 (0.45;1.40)		0.84 (0.49;1.43)	
Brown	1.03 (0.90;1.18)		1.12 (1.00;1.26)		1.11 (1.01;1.22)	
Indigenous	0.91 (0.62;1.34)		1.09 (0.82;1.45)		1.16 (0.94;1.45)	
Years of study		< 0,001**		< 0.001**		< 0.001**
0 year	4.06 (3.08;5.35)		3.79 (3.00;5.17)		1.82 (1.52;2.19)	
1 to 4 years	3.38 (2.60;4.38)		3.24 (2.54;4.14)		1.69 (1.39;2.06)	
5 to 8 years	2.86 (2.07;3.95)		2.84 (2.09;3.86)		1.74 (1.50;2.03)	
9 to 11 years	1.83 (1.26;2.64)		2.04 (1.42;2.92)		1.35 (1.03;1.77)	
12 years or more	Reference		Reference		Reference	
Monthly income (minimum wages)		0.225		0.128		0.001**
Up to 1	1.50 (1.00;2.25)		1.59 (1.10;2.29)		1.42 (1.07;1.89)	
1 to 2	1.45 (0.98;2.17)		1.60 (1.12;2.28)		1.39 (1.06;1.81)	
2 to 3	1.46 (0.97;2.18)		1.69 (1.16;2.46)		1.48 (1.13;1.92)	
3 to 5	1.30 (0.78;2.18)		1.43 (0.96;2.14)		1.17 (0.85;1.61)	
Greater than 5	Reference		Reference		Reference	
Macroregion		0.686		0.239		0.053
North	Reference		Reference		Reference	
North East	0.93 (0.79;1.08)		0.94 (0.82;1.07)		0.96 (0.89;1.03)	
Southeast	0.87 (0.71;1.07)		0.80 (0.66;0.97)		0.79 (0.72;0.88)	
South	0.86 (0.70;1.05)		0.88 (0.76;1.02)		0.93 (0.85;1.01)	
Central-West	1.02 (0.86;1.21)		0.94 (0.81;1.10)		0.93 (0.86;1.01)	
Municipality		0.031		0.238		0.246
Interior	Reference		Reference		Reference	
Capital	0.86 (0.74;0.99)		0.93 (0.83;1.05)		0.96 (0.90;1.03)	

PR: prevalence ratio. CI: confidence interval. *Adjustment for the variables in this table, respecting the hierarchical levels of determination: level 1 - distal (macro-region and municipality), level 2 - intermediate (years of schooling and monthly income), and level 3 (sex, age, and skin color); **Wald test for linear trend.

Severe tooth loss was also more prevalent among women (PR=1.22; 95%CI: 1.08-1.40) and increased with age, reaching a PR of 1.62 (95%CI: 1.23-2.14) at 74 years of age. In turn, individuals with brown skin color (PR=1.12; 95%CI: 1.00-1.26) showed a higher prevalence of severe tooth loss compared to those with white skin color, and individuals with no schooling were 3.79 times more likely to experience tooth loss when compared to those with higher educational attainment (PR=3.79; 95%CI: 3.00-5.17).

Women showed a 20% higher probability of lacking functional dentition when compared to men (PR=1.20; 95%CI 1.11-1.29); among those aged 74 years, the prevalence was 24% higher (PR: 1.24; 95%CI 1.08-1.43) than among those aged 65 years. Individuals with brown skin color (PR=1.11; 95%CI 1.01-1.22) showed a higher prevalence of lacking functional dentition when compared to those with white skin color. In addition, both lower educational attainment (PR=1.82; 95%CI 1.52-2.19) and a monthly income of 2 to 3 minimum wages (PR=1.48; 95%CI 1.13-1.92) were strongly associated with dental condition, consistently increasing the probability of the elderly not having functional dentition.

The macro-region variable was not associated with the studied outcomes.

## DISCUSSION

The findings of this study indicated that individual and socioeconomic factors, especially sex, age, skin color, education, and income, are central determinants of the dental condition of elderly people in Brazil, being associated with worse oral health indicators. Tooth loss is a significant global problem, especially among the elderly, due to the progressive decline in self-care abilities, such as tooth brushing, which favors poor oral conditions^23^.

Considering the prevalence of edentulism in Brazil in the years 2003 (53.34%) and 2010 (53.38%), a reduction was observed in 2023, when the prevalence was 36.48%. This reduction occurred in all Brazilian regions and age groups, as well as among all sexes and self-reported race/skin color groups^24^.

Females had a higher probability of edentulism, severe tooth loss, and lack of functional dentition compared to men, corroborating previous national studies^6^ ^,^ ^25^ ^,^ ^26^. However, this finding represents a paradox, since women tend to use dental services more than men. This phenomenon can be explained by mechanisms of gender and intersectionality, in which greater historical access did not necessarily translate into conservative treatments, but rather into mutilating procedures conditioned by a care model focused on pain and urgency^24^ ^,^ ^27^. Furthermore, recent literature indicates that sexism and structural racism operate cumulatively throughout life, exposing women, especially black and mixed-race women, to care trajectories guided by tooth extraction rather than rehabilitation, which justifies the persistence of these inequalities even after adjustment for income and education^28^.

The results showed that all evaluated tooth losses increased progressively with advancing age, confirming previous investigations^25^ ^,^ ^29^. Tooth loss and oral problems accumulate throughout the life course, leading to worse conditions in old age^30^. The functional decline generally manifested at this stage of life may compromise oral hygiene and increase the risk of developing caries and periodontal disease, the main causes of tooth loss^31^. In addition, the presence of multimorbidity is frequent in the elderly, which may imply a lower chance of having a functional dentition and a greater chance of having severe tooth loss^32^.

The high prevalence of lack of functional dentition observed in this study represents not only a dental problem, but also a marker of systemic frailty, with nutritional and functional implications. Recent Brazilian studies show that the loss of masticatory function increases the risk of physical frailty in community-dwelling older adults, while a greater number of teeth is associated with better physical performance^33^ ^,^ ^34^. Furthermore, prosthetic rehabilitation can improve nutritional status and masticatory efficiency in edentulous older adults, suggesting a potential interruption of the cascade of functional decline^35^. Thus, functional dentition should be understood not only as an aesthetic or comfort indicator, but as an essential component of the intrinsic capacity of the older person, according to the WHO’s *Integrated Care for Older People* (ICOPE) guidelines^36^.

The strong inverse association between education/income and all evaluated outcomes (with a clear dose-response gradient) confirms that these determinants operate not only through material pathways (access to services), but also through psychosocial mechanisms accumulated throughout life, including health literacy, autonomy in care-related decisions, and differential exposure to mutilating versus conservative models of care^37^ ^,^ ^38^. This finding reinforces that oral health policies should be integrated into broader social protection strategies, such as income transfer programs, in order to break the intergenerational cycle of health inequalities.

Our findings of a higher frequency of severe tooth loss and lack of functional dentition among mixed-race individuals, compared to white individuals, confirm the persistence of racial inequalities in oral health in Brazil. National studies indicate that black people have a higher prevalence of edentulism and lower use of dental services, even after adjustment for income and education, suggesting the influence of structural determinants and institutional racism on oral health outcomes^24^ ^,^ ^39^ ^,^ ^40^. In this study, the persistence of the association between mixed-race skin color and worse functional outcomes, even after socioeconomic adjustment, indicates that income is not the only mediator of inequality. Structural racism manifests itself through geographic barriers (territories with lower density of specialized services) and discriminatory care processes, in which black and mixed-race populations accumulate more unmet needs and receive less complex therapies over decades^39^ ^,^ ^41^. Such evidence reinforces that racial equity in oral health requires confronting institutional racism within the Brazilian Unified Health System (SUS), ensuring that access results in equivalent quality of care among groups.

In addition to macroregional disparities, this study identified that residing in inland areas was independently associated with a higher prevalence of edentulism, even after adjustment for income and education. This finding suggests that the geographic barrier operates through structural mechanisms that transcend individual socioeconomic status. National data indicate that, among Brazil’s 5,570 municipalities, only 780 had Dental Specialty Centers (CEOs) by 2015, with a concentration in the Northeast (38.3%) and Southeast (36.2%) regions^42^. This unequal distribution has a direct impact on access to conservative treatments: a study conducted in municipalities without CEOs showed that, among patients referred for endodontic treatment, only 19.8% received the treatment, while 45.5% underwent extraction^43^. In contrast, municipalities that implemented CEOs presented lower proportions of tooth extractions, especially when associated with high coverage of oral health teams, demonstrating that expanding the specialized care network promotes a shift toward conservative practices^44^. This finding reinforces the need for regionalization strategies that ensure not only the presence of oral health teams in primary care, but also equitable access to specialized services in remote regions.

From the perspective of public policies, even with the expansion of access provided by SUS and Brasil Sorridente, barriers still persist that prevent populations in greater vulnerability from achieving acceptable standards of oral health. This points to the need for intersectoral strategies that combine income transfer with specific actions for the promotion, prevention, and rehabilitation of oral health, in order to reduce inequalities^45^.

The observed epidemiological transition, with a reduction in total edentulism from 53% (2010) to 36.5% (2023), but the persistence of a high prevalence of non-functional dentition (72.8%), imposes new challenges for planning oral health care within the SUS. This change in profile indicates that the demand for prosthetic rehabilitation has not decreased; on the contrary, it has become more complex, requiring removable partial dentures, overdentures, and, osseointegrated implants in selected contexts^46^ ^,^ ^47^. Despite the growth in denture production by the SUS over the last decade, supply remains insufficient in relation to population needs and is unevenly distributed across regions^48^ ^,^ ^49^. Furthermore, budget impact analyses indicate that implementing large-scale implant programs would require substantial investments, reinforcing the need for prioritization based on cost-effectiveness and equity^50^. In this context, it is essential that the National Oral Health Policy expand not only the coverage of conventional prostheses in primary care, but also ensure technical training, prosthetic follow-up protocols, and monitoring of post-rehabilitation nutritional and functional outcomes, in order to guarantee that care results in effective gains in quality of life^51^.

This study has limitations. Due to its cross-sectional design, it does not allow establishing causality between the analyzed factors, given the possibility of reverse causality. In addition, part of the information was self-reported, which may introduce recall bias or social desirability bias. Although SB Brasil 2023 was designed to ensure national representativeness, some subgroups, such as indigenous people, quilombola communities, and remote rural populations, may have been underrepresented, limiting the generalizability of the findings. Finally, temporal comparisons with previous editions should be interpreted with caution because of possible methodological changes between the surveys.

Among the strengths of the study are the representative sample and the use of data from the most recent national oral health survey. The methodological rigor, including examiner calibration and validated diagnostic criteria, reinforces the reliability of the data and the relevance of the findings to support public policies aimed at reducing inequalities in oral health.

In conclusion, the evaluated outcomes were more prevalent among women, the oldest older adults, and socially vulnerable groups, highlighting the persistence of important sociodemographic inequalities in the oral health of the Brazilian elderly population. These findings suggest that such inequalities transcend individual factors, reflecting structural barriers that require systemic responses. For the SUS, the implications include the expansion and decentralization of Specialized Dental Centers (CEOs), the incorporation of functional dentition as a monitoring indicator, and the adoption of gender- and race-sensitive clinical protocols. Longitudinal studies are needed to identify critical windows for intervention and to assess the impact of prosthetic rehabilitation on frailty prevention. Equity in oral health remains a central challenge for healthy aging in Brazil.
