# Supplementary material for: Edentulism, severe tooth loss, and lack of functional dentition in elderly people in Brazil: prevalence and social inequalities in the 2023 National Oral Health Survey
Source: Rev Bras Epidemiol. 2026 Jul 24;29(Suppl 1):e260023supl1. doi: 10.1590/1980-549720260023.supl.1 (PMC13403703; doi:10.1590/1980-549720260023.supl.1)
Supplement: Translation

# Translation: Edentulismo, perda dentária severa e ausência de dentição funcional em pessoas idosas no Brasil: prevalência e desigualdades sociais na Pesquisa Nacional de Saúde Bucal 2023

**DOI:** 10.1590/1980-549720260023.supl.1.2

**Authors:** Renato Canevari Dutra da Silva, Heloísa Silva Guerra, Fernanda de Oliveira Meller, Cleidiane Aparecida de Quadra, Elton Brás Camargo, Antônio Augusto Schäfer

**Affiliations:** I Universidade de Rio Verde, Faculdade de Odontologia do Campus Rio Verde, Grupo de Estudos e Pesquisas em Epidemiologia e Saúde - Rio Verde (GO), Brasil.; II Universidade de Rio Verde, Faculdade de Medicina do Campus Goiânia, Grupo de Estudos e Pesquisas em Epidemiologia e Saúde - Goiânia (GO), Brasil.; III Universidade do Extremo Sul Catarinense, Programa de Pós-graduação em Saúde Coletiva - Criciúma (SC), Brasil.; IV Universidade de Rio Verde, Pró-reitora de Pós-Graduação, Grupo de Estudos e Pesquisas em Epidemiologia e Saúde - Rio Verde (GO), Brasil.

**Keywords:** Perda de dente, Idoso, Saúde bucal, Iniquidade social, Epidemiologia

## Abstract

**Objetivo:**

Estimar a prevalência de edentulismo, perda dentária severa e ausência de dentição funcional em idosos brasileiros e analisar desigualdades sociais, raciais e regionais associadas, com base na Pesquisa Nacional de Saúde Bucal 2023.

**Métodos:**

Estudo transversal de base populacional com indivíduos ≥ 65 anos. Os desfechos avaliados foram edentulismo, perda dentária severa (0-8 dentes) e ausência de dentição funcional (< 21 dentes). As associações foram estimadas por regressão de Poisson com variância robusta.

**Resultados:**

Foram incluídos 9.746 idosos. A prevalência de edentulismo foi de 36,5%; de perda dentária severa, 48,1%; e de ausência de dentição funcional, 72,9%. Na análise ajustada, mulheres apresentaram maior prevalência de todos os desfechos, assim como indivíduos mais idosos. A baixa escolaridade destacou-se como principal fator associado às piores condições dentárias: idosos sem estudo apresentaram maior prevalência de edentulismo (RP=3,62; IC95% 2,48-5,30) e perda dentária severa (RP=3,79; IC95% 3,00-5,17), além de maior ausência de dentição funcional (RP=1,82; IC95% 1,52-2,19), em comparação aos mais escolarizados. Idosos de cor de pele parda apresentaram maior prevalência de perda dentária severa e ausência de dentição funcional, enquanto residir em capitais associou-se a menor prevalência de edentulismo.

**Conclusão:**

O edentulismo, a perda dentária severa e a ausência de dentição funcional permanecem fortemente associados a desigualdades sociais, raciais e regionais no Brasil. Os achados reforçam a necessidade de políticas públicas voltadas à equidade em saúde bucal, com ênfase na prevenção, reabilitação protética e ampliação do acesso aos serviços para populações vulneráveis.

## INTRODUÇÃO

A condição da dentição constitui um importante indicador da saúde bucal, refletindo a história cumulativa de exposição a fatores de risco, adoecimento e acesso a serviços odontológicos ao longo da vida. Alterações na dentição influenciam aspectos estéticos, nutricionais e psicossociais, impactando a mastigação, fala, autoestima e qualidade de vida dos indivíduos^1^ ^,^ ^2^. Apesar da redução global na prevalência de alterações dentárias nas últimas décadas, os desafios permanecem em países de baixa e média renda, onde determinantes sociais continuam a moldar a distribuição desigual desses desfechos^3^ ^,^ ^4^.

O edentulismo, caracterizado pela ausência total de dentes, é um importante problema de saúde pública, por refletir a carga acumulada de doenças bucais e profundas desigualdades sociais^5^. Em diferentes contextos brasileiros, estudos apontam prevalências entre 46,4% em amostras domiciliares municipais e mais de 62% em populações institucionalizadas, sugerindo possível estagnação do declínio observado em décadas anteriores em subgrupos específicos^6^ ^,^ ^7^ ^,^ ^8^.

Além do edentulismo, condições intermediárias de redução do número de dentes funcionam como marcadores sensíveis das trajetórias de saúde bucal. A literatura destaca que a manutenção de uma dentição funcional, frequentemente definida por 20 ou 21 dentes, é fundamental para reduzir impactos negativos na qualidade de vida, embora os indicadores funcionais ainda variem entre os estudos nacionais^9^ ^,^ ^10^. O número de dentes remanescentes distribui-se de forma desigual, refletindo diferenças regionais e socioeconômicas e desigualdades no acesso a cuidados preventivos e restauradores^11^ ^,^ ^12^ ^,^ ^13^ ^,^ ^14^.

Indivíduos de menor escolaridade e baixa renda, pertencentes a grupos raciais minorizados, residentes em áreas rurais ou em regiões menos desenvolvidas, e mulheres apresentam maior probabilidade de alterações dentárias significativas^1^ ^,^ ^6^ ^,^ ^12^. Entretanto, nota-se uma lacuna persistente na literatura prévia ao levantamento feito pela Pesquisa Nacional de Saúde Bucal (SB Brasil) 2023 quanto à análise aprofundada das disparidades raciais e à integração de dados de oferta de serviços para explicar as variações regionais observadas, uma vez que a maioria das evidências disponíveis provém de amostras locais ou de conveniência com poder de generalização limitado^15^ ^,^ ^16^.

A Política Nacional de Saúde Bucal - Brasil Sorridente - ampliou o acesso a cuidados preventivos e restauradores, além de oferecer reabilitação protética; porém, persistem desafios relacionados à cobertura, continuidade do cuidado e superação das desigualdades regionais e sociais^17^ ^,^ ^18^ ^,^ ^19^.

O levantamento epidemiológico nacional SB Brasil constitui a principal fonte de dados sobre as condições de saúde bucal da população brasileira. Suas edições anteriores (2003 e 2010) evidenciaram marcadas desigualdades regionais e socioeconômicas em indicadores como perda dentária e edentulismo^20^ ^,^ ^21^.

Diante da defasagem de mais de uma década nos dados nacionais mais abrangentes, o SB Brasil 2023 é essencial para atualizar o panorama da saúde bucal no país e superar limitações de estudos fragmentados. Assim, este estudo objetivou estimar a prevalência de edentulismo, perda dentária severa e ausência de dentição funcional em indivíduos com 65 anos ou mais, bem como avaliar desigualdades sociais, raciais e regionais associadas a esses desfechos, com base em dados representativos do SB Brasil 2023.

## MÉTODOS

### Desenho do estudo

Trata-se de estudo transversal de base populacional, com dados do SB Brasil 2023, coordenado pelo Ministério da Saúde. O SB Brasil é o principal inquérito epidemiológico nacional em saúde bucal, com amostragem probabilística em múltiplos estágios. Neste estudo, analisou-se a população idosa (≥ 65 anos), visando avaliar desfechos relacionados à perda dentária e à manutenção da dentição funcional.

### População e amostragem

O plano amostral do SB Brasil 2023 foi delineado para garantir representatividade nacional, regional, por capitais e interior. A seleção ocorreu em três etapas: setores censitários, sorteados com base no Cadastro Nacional de Endereços para Fins Estatísticos do Instituto Brasileiro de Geografia e Estatística (CNEFE/IBGE); domicílios, selecionados sistematicamente em cada setor; e indivíduos elegíveis, convidados conforme as idades-índice do inquérito (5, 12, 15-19, 35-44 e 65-74 anos). Neste estudo, incluíram-se todos os participantes com 65 anos ou mais, totalizando 9.746 idosos.

### Coleta de dados

A coleta ocorreu entre 2022 e 2023 por equipes formadas por cirurgiões-dentistas e anotadores, previamente treinados e calibrados. O processo incluiu atividades teóricas, exercícios virtuais e etapas presenciais, com análise de fotografias e exames clínicos padronizados.

A concordância com o padrão-ouro foi avaliada pelo coeficiente Kappa, exigindo-se valor mínimo de 0,65 para atuação em campo. Os exames clínicos domiciliares foram realizados com iluminação natural, espelhos bucais planos e sondas comunitárias esterilizadas, segundo critérios da Organização Mundial da Saúde (OMS). Também foi aplicado questionário estruturado com informações demográficas, socioeconômicas, de acesso a serviços de saúde e agravos bucais, incluindo perda dentária, condição dentária, traumatismos e necessidade de tratamento.

### Variáveis estudadas

Foram considerados três desfechos relacionados à saúde bucal: “edentulismo”, definido como a perda total de dentes naturais (sim; não); “perda dentária severa”, que é a presença de 0 a 8 dentes remanescentes (sim; não); e “ausência de dentição funcional”, definida como a presença de 21 ou mais dentes naturais (sim; não). É importante destacar que os três desfechos estudados não são mutuamente excludentes, podendo um mesmo indivíduo enquadrar-se em mais de uma categoria.

As variáveis de exposição incluídas para avaliar desigualdades sociais, raciais e regionais foram: sexo (masculino; feminino), idade (em anos completos), anos de estudo (0; 1-4; 5-8; 9-11; ≥ 12 anos), renda domiciliar mensal em salários mínimos (≤ 1; 1-2; 2-3; 3-5; > 5), cor da pele autorreferida (branca; preta; parda; amarela; indígena), macrorregião de residência (Norte; Nordeste; Sudeste; Sul; Centro-Oeste), município (capital; interior); recebimento de Bolsa Família e/ou outros programas governamentais de transferência de renda (sim; não).

### Análise estatística

Análise descritiva de todas as variáveis estudadas foi apresentada por meio das frequências absolutas e relativas, com seus respectivos Intervalos de Confiança de 95% (IC95%).

Para avaliar as desigualdades sociais, raciais e regionais nos desfechos de saúde bucal estudados foi utilizado o teste qui-quadrado de Pearson, com nível de significância de 5%, para as análises brutas.

Para verificar a independência das associações em relação a potenciais fatores de confusão, realizaram-se análises ajustadas por regressão de Poisson com variância robusta, estimando Razões de Prevalência (RP) e respectivos IC95%. Os valores p apresentados são correspondentes ao teste de Wald para tendência linear. A análise ajustada foi conduzida por meio de modelo hierárquico de determinação, fundamentado no referencial teórico dos determinantes sociais da saúde proposto por Dahlgren e Whitehead (1991). As variáveis contextuais relacionadas ao local de residência (macrorregião e município) foram consideradas no nível distal. No nível intermediário foram incluídas as variáveis socioeconômicas individuais (anos de estudo e renda mensal); no nível proximal, as características demográficas (sexo, idade e cor da pele). A inclusão das variáveis no modelo seguiu essa estrutura hierárquica, respeitando o gradiente teórico de determinação entre os níveis^22^.

Todas as análises incorporaram o peso amostral, o efeito do desenho complexo e a estrutura de estratificação da amostra, por meio do comando *svy* no software *Stata* 17.0 (StataCorp, College Station, Estados Unidos), garantindo a correção para os efeitos do plano amostral.

### Aspectos éticos

Em obediência à Resolução n.º 466, de 12 de dezembro de 2012, do Conselho Nacional de Saúde (CNS), houve a aprovação do Projeto pela Comissão Nacional de Ética em Pesquisa (Conep), em 3 de julho de 2021, sob CAAE 34497120.6.3001.0008 e parecer n.º 4.823.054. O Termo de Consentimento Livre e Esclarecido (TCLE) foi obtido de todos os participantes e representantes legais de participantes vulneráveis.

## Declaração de Disponibilidade de Dados:

Todo o conjunto de dados que dá suporte aos resultados deste estudo está disponível mediante solicitação ao Ministério da Saúde: https://www.gov.br/saude/pt-br/composicao/saps/brasil-sorridente/sb-brasil/dados#:~:text=A%20edi%C3%A7%C3%A3o%20mais%20recente%20da%20Pesquisa%20Nacional,a%20porcentagem%20da%20popula%C3%A7%C3%A3o%20brasileira%20livre%20de

## RESULTADOS

A amostra do estudo compreendeu 9.746 indivíduos com 65 anos ou mais, sendo a maioria do sexo feminino (61,9%) e quase a metade de cor de pele parda (45,6%). Além disso, 37,8% dos idosos tinham até 4 anos de estudo, e cerca da metade deles tinha renda mensal de até 2 salários mínimos (46,3%). A Tabela 1 e a Figura 1 apresentam a descrição da amostra e distribuição da prevalência dos desfechos segundo características demográficas e socioeconômicas.

**Tabela 1.** Descrição da amostra e distribuição da prevalência dos desfechos segundo características demográficas e socioeconômicas. SB Brasil, 2023.

Variáveis	Total		Edentulismo		Perda dentária severa		Ausência de dentição funcional	
	N	%	%	IC95%	%	IC95%	%	IC95%
Sexo (N=9.745)				*p=0,041*		*p=0,038*		*p<0,001*
Masculino	3.710	38,1	33,4	30,0;37,1	44,9	40,1;49,5	67,6	63,4;71,6
Feminino	6.035	61,9	38,5	34,4;42,7	50,2	45,7;54,6	76,3	72,7;79;5
Idade (N=9.736)				*p<0,001*		*p<0,001*		*p=0,004*
65 anos	1.638	16,8	26,7	21,0;33,3	38,6	32,5;45,1	63,4	32,5;45,1
66 anos	1.001	10,3	32,8	26,4;40,0	42,4	35,3;49,8	68,8	35,3;49,8
67 anos	902	9,3	2,7	20,8;34,0	41,9	34,1;50,1	70,9	34,1;50,1
68 anos	932	9,6	36,6	29,6;44,1	47,7	40,1;55,5	71,1	40,1;55,5
69 anos	824	8,5	36,9	29,0;45,5	52,7	44,4;60,9	73,1	44,4;61,0
70 anos	888	9,1	32,8	26,6;39,7	43,7	36,5;51,3	74,5	36,5;51,3
71 anos	672	6,8	44,4	34,4;54,8	57,7	46,7;68,0	84,1	46,7;68,0
72 anos	872	9,0	43,0	35,9;50,3	52,1	44,9;59,2	78,6	44,9;59,2
73 anos	800	8,2	43,3	34,5;52,5	53,7	45,2;62,0	74,5	45,2;62,1
74 anos	1.207	12,4	49,8	42,8;56,9	58,5	51,4;65,2	78,8	51,4;65,2
Cor da pele (N=9.599)				*p=0,255*		*p=0,006*		*p<0,001*
Branca	3.601	37,5	34,5	30,0;39,3	44,3	38,9;49,8	67,9	62,7;72,7
Preta	1.353	14,1	38,2	32,5;44,3	49,8	43,6;56,0	78,0	72,1;83,0
Amarela	129	1,3	25,5	13,6;42,6	37,8	22,5;56,0	62,1	37,7;81,6
Parda	4.466	46,5	38,7	35,5;42,0	52,2	48,3;56,1	77,4	74,4;80,2
Indígena	50	0,6	27,0	7,9;61,5	78,4	52,7;92,2	95,7	83,1;99,0
Anos de estudo (N=8.859)				*p<0,001*		*p<0,001*		*p<0,001*
0 ano	1.240	14,1	54,1	48,8;59,3	66,3	60,8;71,3	85,5	81,3;88,9
1 a 4 anos	2.099	23,7	45,0	39,6;50,6	59,4	52,8;65,7	81,4	75,8;85,9
5 a 8 anos	2.484	28,0	38,0	32,1;44,3	50,2	44,5;56,0	78,1	73,9;81,8
9 a 11 anos	1.376	15,5	24,2	18,3;31,3	34,7	27,1;43,1	60,5	49,5;70,5
12 anos ou mais	1.660	18,7	13,3	10,2;17,1	17,0	13,6;21,2	43,8	38,1;49,6
Renda mensal (salários mínimos) (N=9.746)				*p<0,001*		*p<0,001*		*p<0,001*
Até 1	1.995	20,5	41,2	36,7;45,7	53,0	48,0;57,9	79,6	73,8;84,3
1 a 2	2.510	25,8	37,5	32,6;42,6	50,0	43,9;56,2	75,5	70,2;80,1
2 a 3	1.102	11,3	32,7	26,8;39,2	50,1	41,7;58,5	76,7	71,1;81,4
3 a 5	1.005	10,3	27,3	18,8;37,8	37,2	28,3;47,0	59,8	49,8;69,1
Maior que 5	584	5,9	15,3	10,4;22,0	19,3	13,6;26,8	42,5	32,2;53,5
Não informado	2.550	26,2	41,7	35,6;48,0	51,5	45,5;57,5	75,0	69,4;79,8
Macrorregião (N=9.746)				*p=0,579*		*p=0,105*		*p<0,001*
Norte	2.243	23,0	40,0	35,3;44,8	55,4	49,6;61,2	83,0	77,9;87,2
Nordeste	3.272	33,6	37,6	34,0;41,4	51,9	47,5;56,3	79,8	75,6;83,3
Sudeste	1.458	15,0	35,2	29,6;41,4	44,4	37,2;51,8	66,0	60,1;71,4
Sul	1.425	14,6	35,5	30,2;41,3	48,9	43,6;54,2	77,1	72,0;81,5
Centro-Oeste	1.348	13,8	40,5	35,4;45,7	52,3	46,5;58,0	77,4	72,9;81,3
Município (N=9.746)				*p=0,032*		*p=0,251*		*p=0,268*
Interior	2.815	28,9	37,8	33,9;41,8	48,9	44,0;53,8	73,5	69,4;77,3
Capital	6.931	71,1	32,4	29,4;35,4	45,5	42,6;48,5	70,8	68,2;73,3
Benefício assistencial de prestação continuada (N=9.125)				*p=0,241*		*p=0,149*		*p=0,105*
Não	7.772	85,2	35,1	31,7;38,7	46,6	42,4;50,9	71,7	68,2;75,0
Sim	1.353	14,8	39,0	33,5;44,7	51,8	45,8;57,7	77,2	70,8;82,6
Recebimento de Bolsa Família (N=9.190)				*p=0,004*		*p=0,052*		*p<0,001*
Não	8.034	87,4	34,6	31,3;38,0	46,5	42,2;50,8	71,3	67,7;74,6
Sim	1.156	12,6	46,3	38,8;54,1	55,7	48,0;63,1	84,3	78,8;88,7
Benefício de outro programa do governo (N=9.136)				*p<0,001*		*p<0,001*		*p<0,001*
Não	7.999	87,6	33,4	30,3;36,8	45,3	41,3;49,4	71,1	67,5;74,3
Sim	1.137	12,4	49,9	41,3;58,5	60,9	53,4;68,1	81,8	76,0;86,5

IC: intervalo de confiança; Teste qui-quadrado de Pearson.

**Figura 1.** Prevalência dos desfechos de saúde bucal estudados. SB Brasil, 2023.

A prevalência de edentulismo foi de 36,5%, sendo mais prevalente entre as mulheres (38,5%), em comparação aos homens (33,4%; p=0,041). De forma semelhante, a perda dentária grave foi observada com maior frequência entre mulheres (50,2% versus 44,9%; p=0,038), assim como a ausência de dentição funcional, que também apresentou diferenças significativas segundo o sexo (76,3% feminino versus 67,6% masculino; p<0,001).

A prevalência de edentulismo e a perda dentária grave aumentaram progressivamente com a idade. Indivíduos com 65 anos apresentaram 26,7% de edentulismo e 38,6% de perda dentária grave, enquanto aqueles com 74 anos atingiram 49,8% e 58,5%, respectivamente (p<0,001). A ausência de dentição funcional seguiu padrão semelhante, variando de 63,4% aos 65 anos para 78,8% aos 74 anos (p=0,004).

A cor da pele também se associou a dois desfechos avaliados. A perda dentária grave foi mais frequente entre indivíduos pretos (49,8%) e pardos (52,2%) em comparação aos brancos (44,3%; p=0,006), e a ausência de dentição funcional seguiu tendência semelhante, sendo maior entre a cor de pele preta (78,0%) e parda (77,4%) em relação à branca (67,9%; p<0,001).

A variável anos de estudo apresentou associação inversa com todos os desfechos. Indivíduos sem escolaridade apresentaram prevalências de edentulismo, perda dentária grave e ausência de dentição funcional de 54,1, 66,3 e 85,5%, respectivamente, enquanto aqueles com 12 anos ou mais de estudo apresentaram valores significativamente menores (13,3%, 17% e 43,8%; p<0,001). De forma semelhante, a renda mensal também se associou inversamente aos desfechos: indivíduos com até um salário mínimo apresentaram maior prevalência de edentulismo (41,2%), perda dentária grave (53%) e ausência de dentição funcional (79,6%), enquanto aqueles com renda superior a cinco salários mínimos apresentaram prevalências significativamente menores (15,3, 19,3 e 42,5%; p<0,001).

A ausência de dentição funcional foi mais frequente nas regiões Norte (83%) e Nordeste (79,8%), em comparação às demais macrorregiões do país (p<0,001). No âmbito municipal, o edentulismo foi mais prevalente entre residentes do interior (37,8%) do que entre os da capital (32,4%; p=0,032).

Indivíduos beneficiários de programas governamentais apresentaram maiores prevalências nos três desfechos, em comparação àqueles que não recebiam benefícios. Entre os beneficiários do Bolsa Família, 46,3% apresentaram edentulismo versus 34,6% entre não beneficiários (p=0,004), enquanto a ausência de dentição funcional foi de 84,3% versus 71,3% (p<0,001). Resultados semelhantes foram observados entre beneficiários de outros programas governamentais (p<0,001 para todos os desfechos) (Tabela 1 e Figura 1).

Após ajuste para possíveis fatores de confusão, a maioria das associações foi mantida, e os resultados estão apresentados na Tabela 2. Maior prevalência de edentulismo foi encontrada entre mulheres (RP=1,19; IC95% 1,04-1,36) e idosos de mais idade, atingindo a prevalência mais elevada aos 74 anos (RP=1,96; IC95% 1,51-2,52). Idosos não escolarizados (0 ano de estudo) apresentaram 3,62 vezes mais probabilidade de ter edentulismo do que aqueles com maior escolaridade (12 anos ou mais) (RP=3,62; IC95% 2,48-5,30). Além disso, indivíduos que residiam nas capitais do país tiveram menor probabilidade de ter edentulismo (RP=0,86; IC95% 0,74;0,99).

**Tabela 2.** Análise ajustada* da associação entre os desfechos e as características demográficas e socioeconômicas. SB Brasil, 2023.

Variáveis	Edentulismo		Perda dentária severa		Ausência de dentição funcional	
	RP (IC95%)	Valor p	RP (IC95%)	Valor p	RP (IC95%)	Valor p
Sexo		0,002		0,001		< 0,001
Masculino	Referência		Referência		Referência	
Feminino	1,22 (1,08;1,40)		1,24 (1,09;1,41)		1,20 (1,11;1,29)	
Idade (anos)		< 0,001**		0,012		< 0,001**
65	Referência		Referência		Referência	
66	1,26 (0,93;1,72)		1,13 (0,91;1,40)		1,15 (0,94;1,41)	
67	1,04 (0,74;1,47)		1,13 (0,89;1,44)		1,24 (1,06;1,46)	
68	1,30 (1,01;1,67)		1,20 (1,00;1,46)		1,10 (0,96;1,25)	
69	1,37 (0,96;1,95)		1,32 (1,08;1,61)		1,15 (0,98;1,35)	
70	1,11 (0,80;1,55)		0,99 (0,76;1,29)		1,16 (0,98;1,38)	
71	1,56 (1,17;2,08)		1,31 (1,02;1,68)		1,37 (1,21;1,56)	
72	1,54 (1,15;2,05)		1,30 (1,07;1,57)		1,25 (1,10;1,42)	
73	1,49 (1,06;2,10)		1,45 (1,12;1,85)		1,29 (1,10;1,50)	
74	1,62 (1,23;2,14)		1,33 (1,08;1,64)		1,24 (1,08;1,43)	
Cor da pele		0,681		0,037		0,037
Branca	Referência		Referência		Referência	
Preta	0,97 (0,81;1,18)		0,94 (0,78;1,12)		1,06 (0,93;1,22)	
Amarela	0,64 (0,36;1,14)		0,80 (0,45;1,40)		0,84 (0,49;1,43)	
Parda	1,03 (0,90;1,18)		1,12 (1,00;1,26)		1,11 (1,01;1,22)	
Indígena	0,91 (0,62;1,34)		1,09 (0,82;1,45)		1,16 (0,94;1,45)	
Anos de estudo		< 0,001**		< 0,001**		< 0,001**
0 ano	4,06 (3,08;5,35)		3,79 (3,00;5,17)		1,82 (1,52;2,19)	
1 a 4 anos	3,38 (2,60;4,38)		3,24 (2,54;4,14)		1,69 (1,39;2,06)	
5 a 8 anos	2,86 (2,07;3,95)		2,84 (2,09;3,86)		1,74 (1,50;2,03)	
9 a 11 anos	1,83 (1,26;2,64)		2,04 (1,42;2,92)		1,35 (1,03;1,77)	
12 anos ou mais	Referência		Referência		Referência	
Renda mensal (salários mínimos)		0,225		0,128		0,001**
Até 1	1,50 (1,00;2,25)		1,59 (1,10;2,29)		1,42 (1,07;1,89)	
1 a 2	1,45 (0,98;2,17)		1,60 (1,12;2,28)		1,39 (1,06;1,81)	
2 a 3	1,46 (0,97;2,18)		1,69 (1,16;2,46)		1,48 (1,13;1,92)	
3 a 5	1,30 (0,78;2,18)		1,43 (0,96;2,14)		1,17 (0,85;1,61)	
Maior que 5	Referência		Referência		Referência	
Macrorregião		0,686		0,239		0,053
Norte	Referência		Referência		Referência	
Nordeste	0,93 (0,79;1,08)		0,94 (0,82;1,07)		0,96 (0,89;1,03)	
Sudeste	0,87 (0,71;1,07)		0,80 (0,66;0,97)		0,79 (0,72;0,88)	
Sul	0,86 (0,70;1,05)		0,88 (0,76;1,02)		0,93 (0,85;1,01)	
Centro-Oeste	1,02 (0,86;1,21)		0,94 (0,81;1,10)		0,93 (0,86;1,01)	
Município		0,031		0,238		0,246
Interior	Referência		Referência		Referência	
Capital	0,86 (0,74;0,99)		0,93 (0,83;1,05)		0,96 (0,90;1,03)	

RP: razão de prevalência. IC: intervalo de confiança. *Ajuste para as variáveis dessa tabela, respeitando os níveis hierárquicos de determinação: nível 1 - distal (macrorregião e município), nível 2 - intermediário (anos de estudo e renda mensal) e nível 3 (sexo, idade e cor da pele); **Teste de Wald para tendência linear.

A perda dentária severa também foi mais prevalente entre mulheres (RP=1,22; IC95%: 1,08-1,40) e aumentou com a idade, alcançando RP de 1,62 (IC95% 1,23-2,14) aos 74 anos. Por sua vez, indivíduos de cor de pele parda (RP=1,12; IC95% 1,00-1,26) apresentaram maior prevalência de perda dentária severa em relação aos de cor de pele branca, e aqueles sem escolaridade tiveram 3,79 vezes mais probabilidade de ter perda dentária quando comparados aos mais escolarizados (RP=3,79; IC95% 3,00-5,17).

Mulheres apresentaram uma probabilidade 20% maior de ausência de dentição funcional quando comparadas aos homens (RP=1,20; IC95% 1,11-1,29); já naqueles com 74 anos de idade, a prevalência foi de 24%, maior (RP: 1,24; IC95% 1,08-1,43) do que os de 65 anos. Indivíduos de cor de pele parda (RP=1,11; IC95% 1,01-1,22) apresentaram maior prevalência de ausência de dentição funcional quando comparados aos de cor de pele branca. Além disso, tanto a menor escolaridade (RP=1,82; IC95% 1,52-2,19) quanto a renda mensal de 2 a 3 salários mínimos (RP=1,48; IC95% 1,13-1,92) estiveram fortemente associadas à condição dentária, aumentando de forma consistente a probabilidade de os idosos não terem dentição funcional.

A variável macrorregião não esteve associada aos desfechos estudados.

## DISCUSSÃO

Os achados deste estudo indicaram que fatores individuais e socioeconômicos, especialmente sexo, idade, cor da pele, escolaridade e renda, são determinantes centrais da condição dentária de pessoas idosas no Brasil, associando-se a piores indicadores de saúde bucal. A perda dentária é um problema global relevante, sobretudo entre idosos, em razão do declínio progressivo de habilidades de autocuidado, como a escovação, o que favorece condições bucais precárias^23^.

Considerando a prevalência de edentulismo no Brasil nos anos de 2003 (53,34%) e 2010 (53,38%), observou-se redução em 2023, quando a prevalência foi de 36,48%. Essa redução ocorreu em todas as regiões brasileiras, faixas etárias, além de em todos os sexos e grupos de raça/cor da pele autorreferidos^24^.

O sexo feminino apresentou uma probabilidade maior de edentulismo, perda dentária severa e ausência de dentição funcional em comparação aos homens, corroborando estudos nacionais prévios^6^ ^,^ ^25^ ^,^ ^26^. Contudo, esse achado configura um paradoxo, visto que mulheres tendem a utilizar mais os serviços odontológicos do que homens. Esse fenômeno pode ser explicado por mecanismos de gênero e interseccionalidade, nos quais o maior acesso histórico não se traduziu necessariamente em tratamentos conservadores, mas sim em procedimentos mutiladores condicionados por um modelo de atenção focado na dor e na urgência^24^ ^,^ ^27^. Além disso, a literatura recente aponta que o sexismo e o racismo estrutural operam de forma cumulativa ao longo da vida, expondo mulheres, especialmente as negras e pardas, a trajetórias de cuidado pautadas pela extração dentária em detrimento da reabilitação, o que justifica a persistência dessas desigualdades mesmo após o ajuste por renda e escolaridade^28^.

Os resultados mostraram que todas as perdas dentárias avaliadas aumentaram progressivamente com o avançar da idade, confirmando investigações anteriores^25^ ^,^ ^29^. As perdas e os problemas bucais se acumulam ao longo do curso da vida, levando a condições piores na velhice^30^. O declínio funcional geralmente manifestado nessa fase da vida pode comprometer a higiene bucal e aumentar o risco de desenvolvimento de cáries e doença periodontal, principais causas de perda dentária^31^. Ademais, é frequente em idosos a presença da multimorbidade, podendo implicar em menor chance de ter uma dentição funcional e maior de ter perda dentária grave^32^.

A alta prevalência de ausência de dentição funcional observada neste estudo representa não apenas um problema odontológico, mas também um marcador de fragilidade sistêmica, com implicações nutricionais e funcionais. Estudos brasileiros recentes mostram que a perda da função mastigatória aumenta o risco de fragilidade física em idosos comunitários, enquanto maior número de dentes associa-se a melhor desempenho físico^33^ ^,^ ^34^. Além disso, a reabilitação protética pode melhorar o estado nutricional e a eficiência mastigatória em idosos edêntulos, sugerindo potencial interrupção da cascata de declínio funcional^35^. Assim, a dentição funcional deve ser compreendida não apenas como indicador estético ou de conforto, mas como componente essencial da capacidade intrínseca da pessoa idosa, conforme as diretrizes *Integrated Care for Older People* (ICOPE) da OMS^36^.

A forte associação inversa entre escolaridade/renda e todos os desfechos avaliados (com gradiente dose-resposta evidente) confirma que esses determinantes operam não apenas por vias materiais (acesso a serviços), mas também por mecanismos psicossociais acumulados ao longo da vida, incluindo letramento em saúde, autonomia nas decisões de cuidado e exposição diferencial a modelos assistenciais mutilatórios versus conservadores^37^ ^,^ ^38^. Esse achado reforça que políticas de saúde bucal devem ser integradas a estratégias de proteção social mais amplas, como os programas de transferência de renda, para romper o ciclo intergeracional de desigualdades em saúde.

Nossos achados de maior frequência de perda dentária grave e de ausência de dentição funcional entre indivíduos pardos, em comparação aos brancos, confirmam a persistência das desigualdades raciais na saúde bucal no Brasil. Estudos nacionais apontam que pessoas negras apresentam maior prevalência de edentulismo e menor uso de serviços odontológicos, mesmo após ajuste por renda e escolaridade, sugerindo a influência de determinantes estruturais e do racismo institucional sobre os desfechos bucais^24^ ^,^ ^39^ ^,^ ^40^. Neste estudo, a persistência da associação entre cor da pele parda e piores desfechos funcionais, mesmo após o controle socioeconômico, indica que a renda não é o único mediador da desigualdade. O racismo estrutural manifesta-se por meio de barreiras geográficas (territórios com menor densidade de serviços especializados) e processos assistenciais discriminatórios, nos quais populações negras e pardas acumulam mais necessidades não atendidas e recebem terapias menos complexas ao longo de décadas^39^ ^,^ ^41^. Tais evidências reforçam que a equidade racial na saúde bucal exige o enfrentamento do racismo institucional no Sistema Único de Saúde (SUS), garantindo que o acesso resulte em qualidade de cuidado equivalente entre os grupos.

Além das disparidades macrorregionais, este estudo identificou que residir no interior esteve independentemente associado a maior prevalência de edentulismo, mesmo após ajuste por renda e escolaridade. Esse achado sugere que a barreira geográfica opera por mecanismos estruturais que transcendem a condição socioeconômica individual. Dados nacionais indicam que, dos 5.570 municípios brasileiros, apenas 780 possuíam Centros de Especialidades Odontológicas (CEOs) até 2015, com concentração nas regiões Nordeste (38,3%) e Sudeste (36,2%)^42^. Essa distribuição desigual tem impacto direto sobre o acesso a tratamentos conservadores: estudo em municípios sem CEO mostrou que, dos pacientes encaminhados para endodontia, apenas 19,8% receberam o tratamento, e 45,5% foram submetidos à extração^43^. Em contrapartida, municípios que implantaram CEOs apresentaram menores proporções de exodontias, especialmente quando associados a alta cobertura de equipes de saúde bucal, evidenciando que a ampliação da rede especializada favorece o deslocamento para práticas conservadoras^44^. Tal achado reforça a necessidade de estratégias de regionalização que garantam não apenas a presença de equipes de saúde bucal na atenção primária, mas também o acesso equitativo a serviços especializados em regiões remotas.

Do ponto de vista das políticas públicas, mesmo com a ampliação do acesso proporcionada pelo SUS e pelo Brasil Sorridente, ainda persistem barreiras que impedem que populações em maior vulnerabilidade alcancem padrões aceitáveis de saúde bucal. Isso aponta para a necessidade de estratégias intersetoriais que aliem a transferência de renda a ações específicas de promoção, prevenção e reabilitação em saúde bucal, de forma a reduzir iniquidades^45^.

A transição epidemiológica observada, com redução do edentulismo total de 53% (2010) para 36,5% (2023), mas manutenção de alta prevalência de dentição não funcional (72,8%), impõe novos desafios ao planejamento da atenção em saúde bucal no SUS. Essa mudança de perfil indica que a demanda por reabilitação protética não diminuiu; pelo contrário, tornou-se mais complexa, exigindo próteses parciais removíveis, *overdentures* e, em contextos selecionados, implantes osseointegrados^46^ ^,^ ^47^. Apesar do crescimento na produção de próteses pelo SUS na última década, a oferta permanece insuficiente ante às necessidades populacionais e desigualmente distribuída entre as regiões^48^ ^,^ ^49^. Além disso, análises de impacto orçamentário indicam que a implementação de programas de implantes em larga escala demandaria investimentos substanciais, o que reforça a necessidade de priorização baseada em custo-efetividade e equidade^50^. Nesse contexto, é fundamental que a Política Nacional de Saúde Bucal amplie não apenas a cobertura de próteses convencionais na atenção primária, mas também garanta capacitação técnica, protocolos de seguimento protético e monitoramento de desfechos nutricionais e funcionais pós-reabilitação, de modo a assegurar que o cuidado resulte em ganhos efetivos de qualidade de vida^51^.

Este estudo apresenta limitações. Por seu delineamento transversal, não permite estabelecer causalidade entre os fatores analisados, diante da possibilidade de causalidade reversa. Além disso, parte das informações foi autorreferida, o que pode introduzir viés recordatório ou de desejabilidade social. Embora o SB Brasil 2023 tenha sido planejado para garantir representatividade nacional, alguns subgrupos, como indígenas, quilombolas e populações rurais remotas, podem ter sido sub-representados, limitando a generalização dos achados. Por fim, comparações temporais com edições anteriores devem ser interpretadas com cautela, em razão de possíveis mudanças metodológicas entre os levantamentos.

Entre os pontos fortes do estudo, destacam-se a amostra representativa e o uso de dados do mais recente inquérito nacional de saúde bucal. O rigor metodológico, com calibração dos examinadores e critérios diagnósticos validados, reforça a confiabilidade dos dados e a relevância dos achados para subsidiar políticas públicas de redução das iniquidades em saúde bucal.

Em conclusão, os desfechos avaliados foram mais prevalentes entre mulheres, idosos mais velhos e grupos socialmente vulnerabilizados, evidenciando a persistência de importantes desigualdades sociodemográficas na saúde bucal da população idosa brasileira. Esses achados sugerem que tais desigualdades transcendem fatores individuais, refletindo barreiras estruturais que exigem respostas sistêmicas. Para o SUS, as implicações incluem a expansão e interiorização dos CEOs, a incorporação da dentição funcional como indicador de monitoramento e a adoção de protocolos clínicos sensíveis a gênero e raça. Estudos longitudinais são necessários para identificar janelas críticas de intervenção e avaliar o impacto da reabilitação protética na prevenção da fragilidade. A equidade em saúde bucal permanece um desafio central para o envelhecimento saudável no Brasil.
